# Combined Metabolomics and Network Pharmacology to Reveal Anti-Diabetic Mechanisms and Potential Pharmacological Components of *Synsepalum dulcificum*

**DOI:** 10.3390/plants14142132

**Published:** 2025-07-10

**Authors:** Yong Huang, Shiyu Wang, Rong Ding, Shaohua Wu

**Affiliations:** Key Laboratory for Southwest Microbial Diversity of the Ministry of Education, Yunnan Institute of Microbiology, School of Life Sciences, Yunnan University, Kunming 650500, China; hybb12580@163.com (Y.H.); 17787947998@163.com (S.W.); dr08221104@163.com (R.D.)

**Keywords:** *Synsepalum dulcificum*, widely targeted metabolomics, network pharmacology, anti-diabetic, α-glucosidase inhibitory

## Abstract

The plant *Synsepalum dulcificum* is notable for its considerable edible and medicinal value, with a longstanding history as a folk remedy for diabetes. Its chemical constituents are rich and structurally diverse. However, there is limited information regarding the metabolic basis of these characteristics, and the biological activities and mechanisms underlying its blood glucose-lowering effects remain incompletely understood. In this study, we conducted a widely targeted metabolomics analysis of the stems, leaves, and fruits of *S. dulcificum* using UPLC-ESI-MS/MS to compare the differences in metabolite profiles among these three tissue types. Our analysis identified a total of 2544 secondary metabolites, primarily consisting of flavonoids and triterpenes, categorized into thirteen distinct compound classes. We selected differential metabolites through multivariate statistical analysis, revealing significant differences among the metabolite profiles of the three tissue types, with flavonoids being the most abundant compounds. Furthermore, we investigated the anti-diabetic mechanisms and potential pharmacological components of *S. dulcificum* utilizing network pharmacology and molecular docking techniques. Finally, the α-glucosidase inhibitory activity of the potential active components was evaluated using in vitro experiments. These findings establish a foundation for the future application of *S. dulcificum* in the prevention and treatment of diabetes.

## 1. Introduction

Diabetes mellitus (DM) is characterized by hyperglycemia, which involves relative insulin deficiency, insulin resistance (IR), or both [1]. Chronic hyperglycemia caused by diabetes can lead to irreversible damage and dysfunction in various tissues, particularly in the eyes, kidneys, heart, blood vessels, and nerves [2]. Type 2 diabetes mellitus (T2DM) accounts for 90% of diabetes cases; overall, diabetes has become the third largest non-communicable disease after cardiovascular diseases and cancer, imposing a significant social and economic burden and posing a serious threat to global human health [3]. Currently, the main treatments for type 2 diabetes include insulin and various oral hypoglycemic agents, such as α-glucosidase inhibitors, sulfonylureas, metformin, and thiazolidinediones [4]. However, these drugs have significant side effects, including hypoglycemia, weight gain, and increased gastrointestinal issues [5]. Therefore, the targeted identification of α-glucosidase inhibitors and insulin resistance modulators derived from food and medicinal plants may provide a safer and less toxic approach to treating diabetes patients, potentially representing an effective method [6].

*Synsepalum dulcificum* (SD), a kind of perennial evergreen shrub, belonging to the genus *Synsepalum* within the family *Sapotaceae*, is indigenous to the tropical regions of West and Central Africa. Notably, this plant is celebrated for the capacity of its fruit to transform sour flavors into sweet ones, which is the origin of its moniker “miracle fruit” [7]. The fruit resembles coffee beans in size and contains relatively large seeds enveloped in translucent flesh [8]. Miracle fruit demonstrates slow growth and possesses a long lifespan. Current research has identified various chemical compositions in different tissues of SD, including miraculin, alkaloids, phytosterols, triterpenes, flavonoids, amino acids, and vitamins. Furthermore, scientific investigations have established a range of pharmacological properties associated with miracle fruit, such as anti-diabetic, cholesterol-lowering, anti-hyperuricemic, antioxidant, anticonvulsant, and anti-cancer activities [9,10].

For instance, Wang et al. [11] reported that (+)-syringaresinol and (+)-epi-syringaresinol, isolated from the stem of SD, demonstrated superior DPPH radical scavenging activity compared to the positive control, vitamin C [11]. In addition to its antioxidant properties, the hypoglycemic potential of SD has garnered considerable interest. Furthermore, Obafemi et al. [12] evaluated the anti-diabetic activity of the leaf extract in type 2 diabetic albino rats and found that both the leaf extract and the positive control, glibenclamide, significantly lowered blood glucose levels in the diabetic subjects. Huang et al. [13] demonstrated that both leaf and seed extracts could inhibit the elevation of plasma total cholesterol in disease-affected mice. Further analysis led to the isolation of two triterpenes from the seed ethanol extract, which were the primary compounds responsible for the observed cholesterol-lowering activity. Existing research on SD predominantly concentrates on the pharmacological effects of the crude extract and traditional isolation of chemical constituents. There is a lack of research on the specific active mechanisms of secondary metabolites from SD, particularly concerning the widely circulated anti-diabetic activities in folk practices. To gain a comprehensive understanding of potential pharmacological ingredients of the plant, it is crucial to conduct an in-depth investigation on the differences of chemical composition among respective tissues of SD.

Metabolomics is an advanced technology for revealing physiological and bio-chemical processes by either targeting or non-targeting detection of metabolites, commonly used to characterize the chemical compositions of natural products as well as the variations in the types and contents of metabolites in organisms exposed to stress [14]. Metabolomics has been extensively employed to identify hypoglycemic agents and elucidate the mechanisms underlying anti-diabetic effects. For instance, Zhao et al. [15] characterized the energy metabolism profiles of patients with diabetic peripheral neuropathy (DPN) and rat models utilizing targeted metabolomics technology based on HPLC-MS. The study demonstrated that the traditional Chinese medicine JMT activates AMP-activated protein kinase (AMPK) and restores energy metabolism homeostasis, thus exerting a neuroprotective effect on peripheral nerves.

Network pharmacology is a method for effectively predicting relevant targets and pathways for drug treatment of diseases to further elucidate the underlying mechanisms [16]. Adopting a multi-component synergistic perspective, network pharmacology elucidates potential mechanisms of action and offers novel insights for the investigation of plant materials. For instance, to investigate the mechanism by which the active components of resveratrol exert their effects against diabetic nephropathy, Chen et al. [17] employed network pharmacology, molecular docking, and experimental validation techniques to analyze the interactions between resveratrol and the pathophysiological damage associated with diabetic nephropathy. Nevertheless, to utilize metabolomics and network pharmacology for studying the anti-diabetic mechanisms of secondary metabolites from SD still remains limited.

This study systematically analyzed the differences in metabolites among the stem, leaf, and fruit tissues of SD through the combined metabolomics and network pharmacology. We compared and explained the key pharmacological substances for diabetes prevention and treatment in different parts of SD. By integrating molecular docking and bioinformatics annotation methods, this research could elucidate the differences in pharmacological properties, significantly enhancing the field of medicinal resource development for SD and promoting its utilization against complex metabolic diseases.

## 2. Results

### 2.1. Qualitative Analysis of Secondary Metabolites in Different Parts of SD

To comprehensively determine the characteristics of secondary metabolites, we developed a widely targeted metabolite analysis method based on UPLC-MS/MS to analyze three tissue parts of SD: stems, leaves, and fruits (S, L, and F). Figure S2 shows the total ion current (TIC) overlap display and the multi-peak detection graph for the QC samples. The extremely high overlap rate of the TIC curves observed among various quality control samples provides strong evidence for the reproducibility and reliability of our assessment results (Figure S2A,B). In the multi-peak MRM metabolite detection graph (Figure S2C,D), each detected metabolite is represented by a mass spectral peak of a different color.

**Figure 1:**
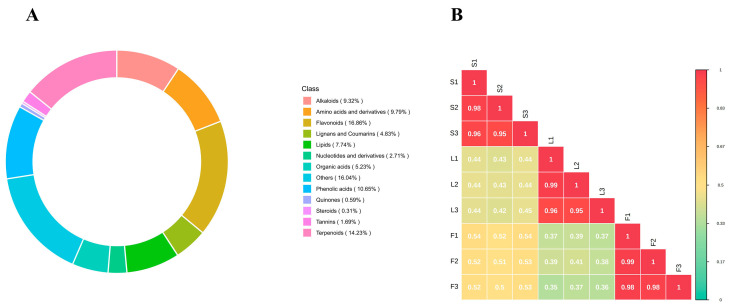
(**A**) Classification of the 2544 secondary metabolites from the stems, leaves, and fruits tissues of SD. (**B**) Correlation analysis.

A total of 2544 secondary metabolites were screened from the SD stems, leaves, and fruits, which can be categorized into thirteen classes: flavonoids (429, 16.86%), terpenoids (362, 14.23%), phenolic acids (271, 10.65%), amino acids and derivatives (249, 9.79%), alkaloids (237, 9.32%), lipids (197, 7.74%), organic acids (133, 5.23%), lignans and coumarins (123, 4.83%), nucleotides and derivatives (69, 2.71%), tannins (43, 1.69%), quinones (15, 0.59%), steroids (8, 0.31%), and others (408, 16.04%) (Figure 1A, Table S1). All thirteen types of secondary metabolites detected in the samples exhibited significant differences in relative levels. The correlation analysis of secondary metabolites in samples from different tissue sites of SD is illustrated in Figure 1. The characteristics between S and L were relatively similar (0.42 ≤|r|≤ 0.45), the secondary metabolite patterns between S and F were relatively similar (0.5 ≤|r|≤ 0.54), and the characteristics between L and F showed a relatively similar value (0.35 ≤|r|≤ 0.41). Among the identified secondary metabolites, flavonoids have the highest content in the stems, leaves, and fruits of SD, primarily composed of flavones (252) and flavonols (177). It is known that they exhibit various biological activities, including antioxidant stress, anti-inflammatory effects, blood glucose-lowering properties, and anti-diabetic activities [18,19]. Terpenoids are the second most important category of compounds, also exhibiting various activities such as antioxidant, anti-inflammatory, and anti-tumor effects [20]. Additionally, in plants, several non-flavonoid metabolites, such as phenolic acids, organic acids, alkaloids, lipids, tannins, quinones, and vitamins, have been shown to have significant health-promoting effects for humans [21,22].

### 2.2. Multivariate Statistical Analysis of Secondary Metabolites

**Figure 2:**
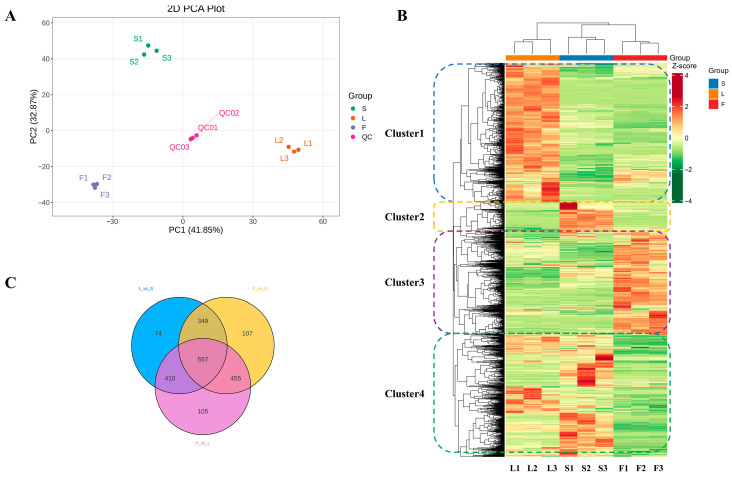
Multivariate statistical analysis of metabolites in SD stems, leaves, and fruits. (**A**) Principal component analysis of S, L, and F metabolites. (**B**) Hierarchical clustering analysis (HCA) of secondary metabolites. (**C**) Venn diagram of metabolite distribution after pairwise grouping of different tissues.

To investigate the overall metabolic differences among various organizational sites of SD as well as the degree of variation between biological replicates, a 2D PCA analysis was performed on the samples (Figure 2A). In the PCA score plot, the two principal components extracted (PC1 and PC2) accounted for a cumulative variance of 74.72%, with PC1 contributing 41.85% and PC2 contributing 32.87%. The results indicated that the stems, leaves, and fruits organizational sites were clearly separated, and the three biological replicates of the same tissue type were tightly clustered together. Samples from different tissues were distinctly separated due to the effects of PC1 and PC2. Moreover, all samples fell within a 95% confidence interval, indicating that there were significant differences in this experiment.

In addition, hierarchical clustering analysis (HCA) was conducted based on the relative content of secondary metabolites, as shown in Figure 2B. The heatmap HCA clearly indicated that the secondary metabolites could be effectively divided into four subgroups: cluster 1, which has the highest levels detected in L; clusters 2 and 4, which have the highest levels detected in S; and cluster 3, which has the highest levels detected in F. Consistent with the PCA results, the findings demonstrate that there are significant differences in the profiles of secondary metabolites among different tissue regions. The Venn diagram analysis of substances contained in different tissue sites of SD shows that there are significant differences in the presence of 2544 compounds across the various tissue sites. Among them, 557 metabolites are shared by S, L, and F; 74 metabolites are unique to L and S; 107 metabolites are unique to F and S; and 105 metabolites are unique to F and L (Figure 2C).

### 2.3. Differences in Metabolic Products of SD in Different Tissue Locations

**Figure 3:**
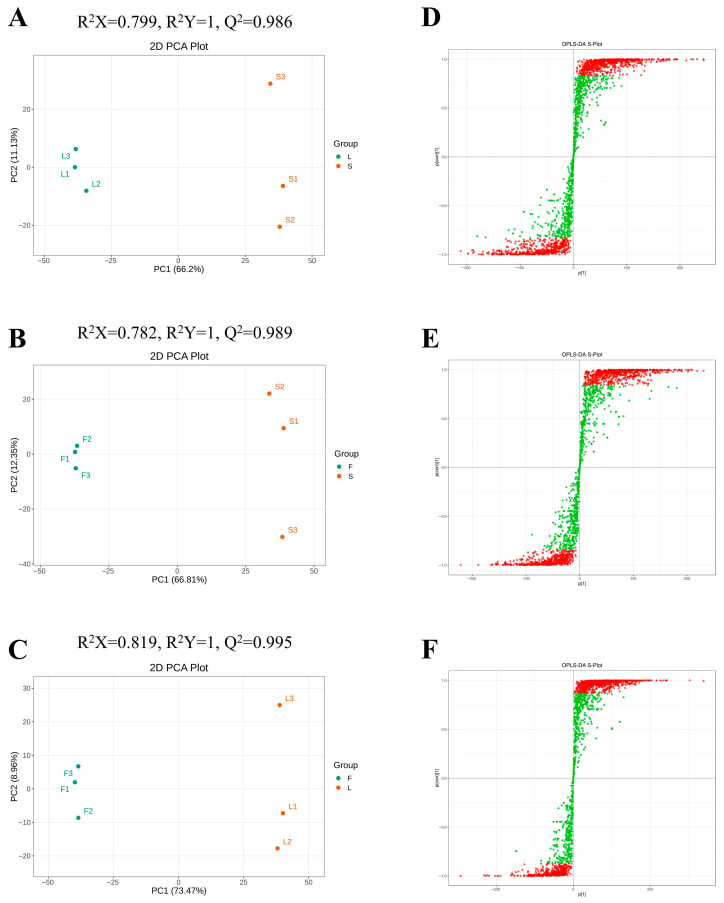
Orthogonal partial least squares discriminant analysis (OPLS-DA) scores. The scores of the OPLS-DA model are presented as (**A**) L vs. S; (**B**) F vs. S; (**C**) F vs. L. OPLS-DA s-plot models are shown as (**D**) L vs. S; (**E**) F vs. S; (**F**) F vs. L. The R^2^Y score and Q^2^ value indicate the explanatory power and predictive ability of the model for the Y matrix. A model can be considered effective when Q^2^ > 0.5, and it is deemed excellent when Q^2^ > 0.9.

Differential analysis was conducted on the substances detected in all samples, with compounds defined as Differentially Abundant Metabolites (DAM) if the fold change (FC) ≥ 2 or FC ≤ 0.5 and the OPLS-DA VIP value ≥ 1 [23]. We further screened the differential metabolites between the pairwise comparisons of S, L, and F using OPLS-DA to evaluate S and L (R2X = 0.799, R2Y = 1, Q2 = 0.986), S and F (R2X = 0.782, R2Y = 1, Q2 = 0.989), and L and F (R2X = 0.819, R2Y = 1, Q2 = 0.995) (Figure 3A–C). The Q2 and R2Y values exceeded 0.9, indicating that the model has good stability and reliability. The OPLS-DA score plots demonstrate that the secondary metabolites from these three tissue regions are well separated in pairs, indicating significant differences in metabolic types (Figure 3D–F). The OPLS-DA score plot indicates a good pairwise separation among the three tissues, indicating that there are differences in the metabolic profiles of these three tissues.

### 2.4. Screening, Functional Annotation, and Enrichment Analysis of Differential Metabolites in Three Tissues of SD

**Figure 4:**
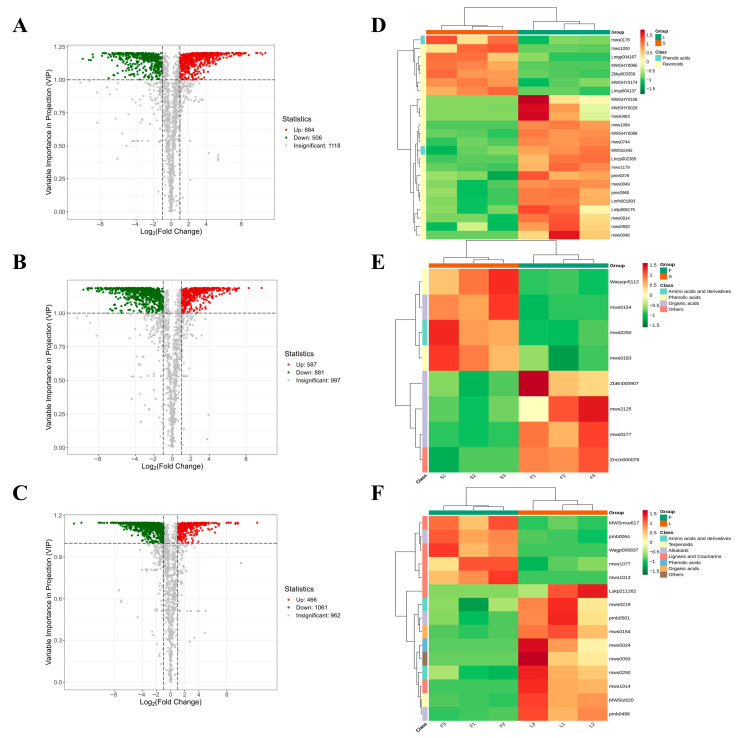
The volcano plots and hierarchical clustering analysis heat maps of differential metabolites for the three comparison groups (L vs. S, F vs. S, F vs. L). (**A**–**C**) The volcano plots for the different pairwise comparisons of differential metabolites are as follows: (**A**) L vs. S; (**B**) F vs. S; (**C**) F vs. L. Red indicates upregulation, green indicates downregulation, and gray indicates no significance. (**D**–**F**) Hierarchical clustering analysis (HCA) heat maps are shown for the respective comparisons: (**A**) L vs. S; (**B**) F vs. S; (**C**) F vs. L. The horizontal axis displays sample names, while the right vertical axis displays the names of the differential metabolites. The deeper the red color, the higher the metabolite content; the deeper the green color, the lower the metabolite content.

Differential metabolites were screened using the criteria of VIP ≥ 1 and fold change ≥ 2 or ≤0.5, and were visualized using a volcano plot (Figure 4A–C). The L vs. S group had fewer differential metabolites, totaling 1390 (884 upregulated and 506 downregulated). In contrast, the F vs. S group had 1468 differential metabolites (587 upregulated and 881 downregulated), while the F vs. L group had the highest number of differential metabolites at 1527 (466 upregulated and 1061 downregulated). The differential metabolites in the L vs. S group were primarily flavonoids, terpenoids, and phenolic acids, such as 6-methoxykaempferol-3-O-glucoside and blumenol C glucoside (Table S2). The differential metabolites in the F vs. S group mainly consisted of lipids, alkaloids, and organic acids, such as dehydrosphingomyelin and tetrandrine (Table S2). Meanwhile, the differential metabolites in the F vs. L group were predominantly nucleotides and their derivatives, lignans, and coumarin compounds, such as violaptene and L-glutamic acid (Table S2). These results strongly indicate that there are significant differences in the composition and regulation of secondary metabolites among the various tissue sites.

Using the annotation information of the differential metabolites identified based on screening criteria, we selected significantly enriched metabolic pathways and performed hierarchical clustering analysis on all differential metabolites within these pathways. This approach aims to better investigate the variation patterns of substance contents in potential important metabolic pathways across different groups (Figure 4D–F). In the “flavonoid biosynthesis (ko00941)” pathway, the contents of chlorogenate (mws0178) and fustin (mws1000) in S were significantly higher than those in L, which may be related to the anti-inflammatory and antioxidant activities of flavonoid compounds [24]. In the “phenylalanine, tyrosine and tryptophan biosynthesis (ko00400)” pathway, shikimate 3-phosphate (ZbBn000907) and phosphoenolpyruvate (mws215) in F were more abundant than in S, which may be associated with organic acids acting as metabolites that sensitize changes in internal pH to exert anti-inflammatory effects [25]. In the “biosynthesis of various plant secondary metabolites (ko00999)” pathway, the contents of secoisolariciresinol (Lskp211262) and L-Aspartate (mws0219) in L were higher than those in F, potentially linking to the antioxidant activity of coumarins [26].

### 2.5. KEGG Annotation and Enrichment Analysis of Differential Metabolites

**Figure 5:**
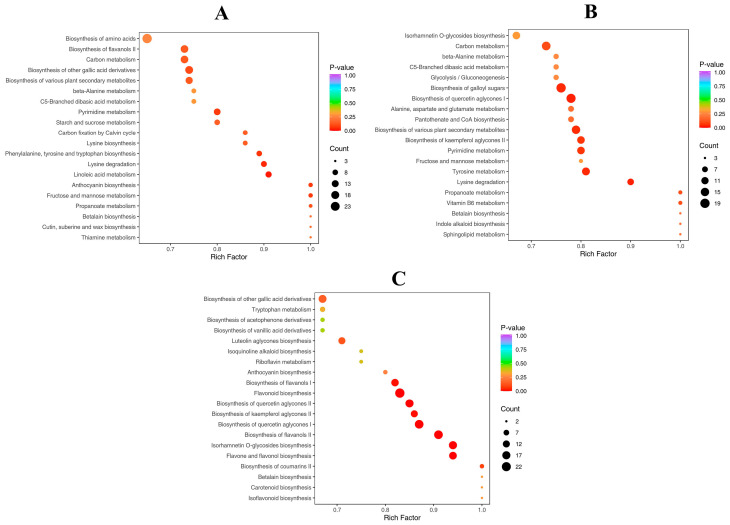
Bubble chart of enriched metabolic pathways for different comparison groups. (**A**) F vs. S. (**B**) F vs. L. (**C**) L vs. S.

To explore the differential metabolite information among L, S, and F, we used the KEGG database for functional annotation and enrichment analysis of the differential metabolites. The bubble plot illustrates the main biological pathways in which the metabolites are involved (Figure 5). Notably, in the L vs. S group, the differential abundant metabolites (DAMs) were primarily enriched in the “flavonoid biosynthesis” and “isorhamnetin O-glycosides biosynthesis” metabolic pathways (*p*-value < 0.05); in the F vs. S group, the DAMs were mainly enriched in the “linoleic acid metabolism” and “Phenylalanine, tyrosine and tryptophan biosynthesis” metabolic pathways (*p*-value < 0.05); in the F vs. L group, the DAMs were mainly enriched in the “Biosynthesis of various plant secondary metabolites” and “biosynthesis of galloyl sugars” metabolic pathways (*p*-value < 0.05). These results indicate that the three organizational regions of SD may have different metabolite profiles, which could be related to their adaptation to the environment and the differing functions of the tissues.

### 2.6. Network Pharmacology Analysis

#### 2.6.1. Screening of DM-Related and SD-Related Targets

**Figure 6:**
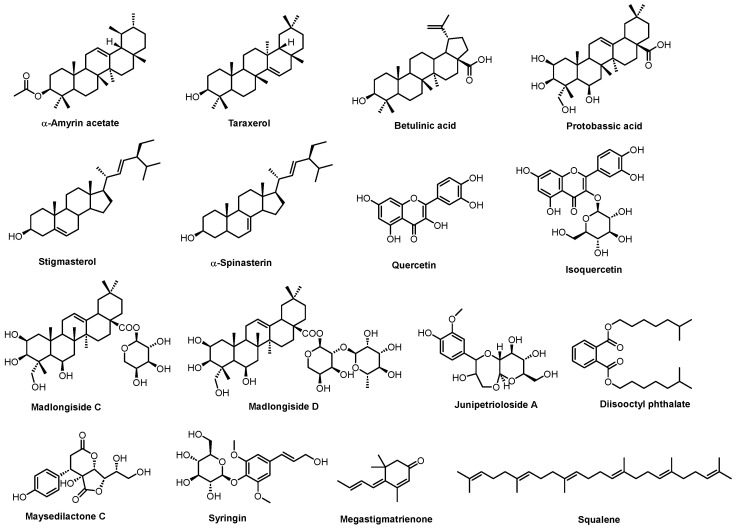
Bioactive compounds of SD.

By integrating extensive targeted metabolomics, the TCMSP database, the pkCSM database, published literature [27,28,29,30], and prior purification of chemical constituents in our laboratory [31], we successfully isolated and identified many secondary metabolites with significant biological activities. Among them, 16 potential bioactive components with favorable pharmacokinetic characteristics and a connection to diabetes disease targets were selected (Figure 6, Table S3)

**Figure 7:**
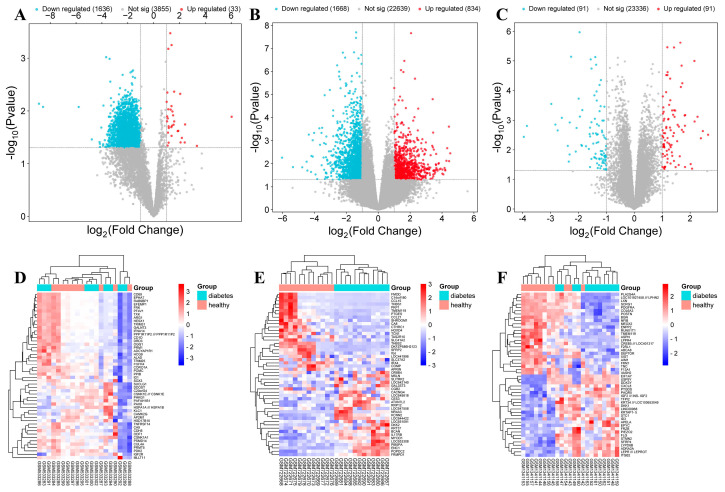
Volcano plots of DEGs between normal group samples and disease group samples in three GSE microarray datasets. (**A**) GSE21340, (**B**) GSE29221, and (**C**) GSE55650. Red represents upregulated genes and blue represents downregulated genes. Heatmaps of the top 50 DEGs sorted by *p* value in three GSE microarray datasets. (**D**) GSE21340, (**E**) GSE29221, and (**F**) GSE55650.

Through differential analysis, 33, 834, and 91 upregulated genes were obtained from the GSE21340, GSE29221, and GSE55650 microarray datasets, respectively (Table S4). Meanwhile, 1636, 1668, and 91 downregulated genes were also identified. The volcano plots of the differentially expressed genes from the three GEO microarray datasets are shown in Figure 7A–C. Additionally, a heatmap of the top 50 differentially expressed genes sorted by *p* value from the three GEO microarray datasets is presented in Figure 7D–F. Furthermore, DM-related protein targets were retrieved using the keyword “diabetes mellitus” from multiple databases such as GeneCard and DisGeNET. After standardizing the targets and removing duplicates, a total of 2447 DM-related protein targets were obtained, among which 603 were found to overlap with the upregulated genes identified from the GEO microarray datasets (Table S5).

**Figure 8:**
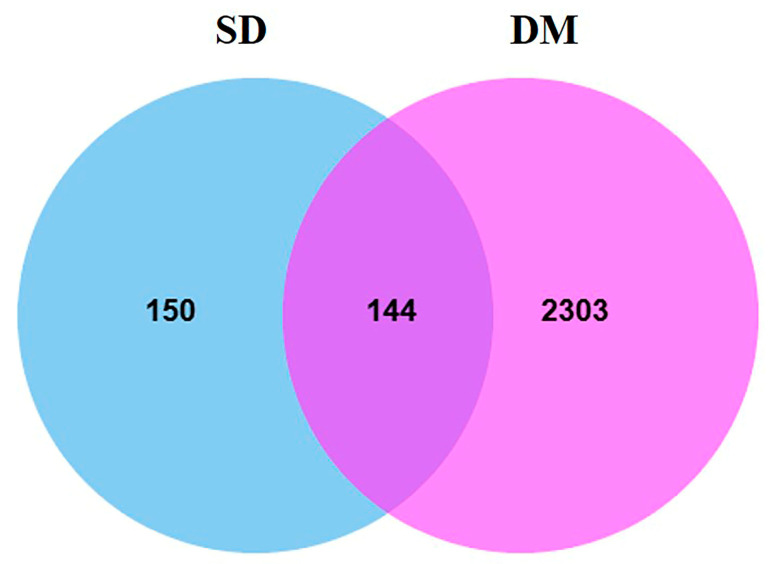
Venn diagram of related targets of sixteen potential active ingredients of SD and DM.

The collected 295 SD-related protein targets were integrated with DM-related targets, and finally, Venn analysis was conducted using a microbioinformatics plat-form (https://www.bioinformatics.com.cn/), revealing 144 potential therapeutic targets for DM (Figure 8).

#### 2.6.2. PPI Analysis of Core Targets

**Figure 9:**
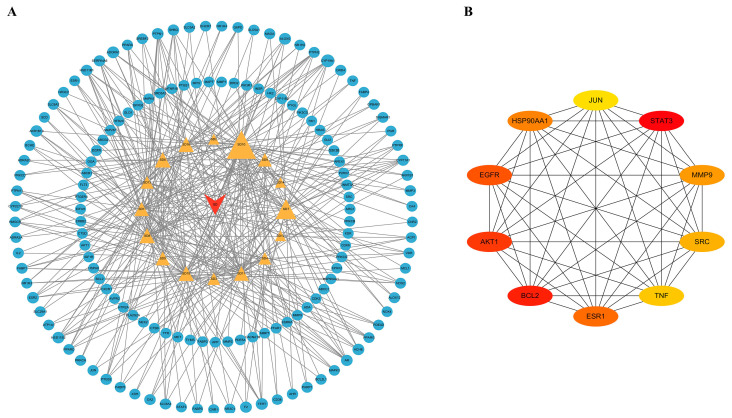
(**A**) PPI network of SD–compounds–targets. The red triangle represents SD, the yellow triangles represent 16 potential effective compounds, and the blue ovals represent potential therapeutic targets. (**B**) Network of the top ten core therapeutic targets.

By inputting 144 candidate therapeutic targets into the STRING database, a PPI network was established. The network was visualized and analyzed for correlation using Cytoscape, where the size and color of the nodes represent their respective degree values (Figure 9A). The CytoNCA plugin was utilized to analyze the topological parameters of the PPI network, ranking the potential active components based on their degree values. Additionally, the top ten core targets were identified, which play a crucial role in the treatment of DM. The core PPI network diagram is depicted in Figure 9B.

#### 2.6.3. GO and KEGG Pathway Enrichment Analyses

**Figure 10:**
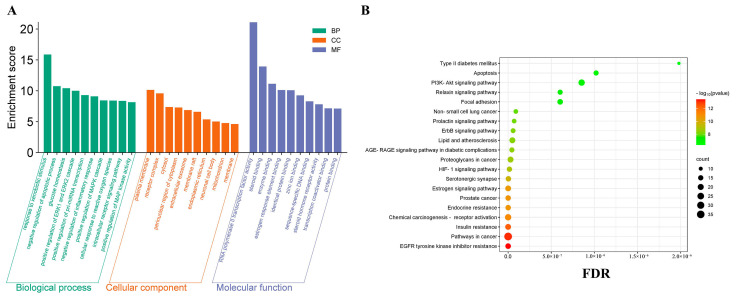
(**A**) GO enrichment analysis of the top 10 GO functional terms. (**B**) KEGG pathway enrichment analysis of the top 20 pathways.

**Figure 11:**
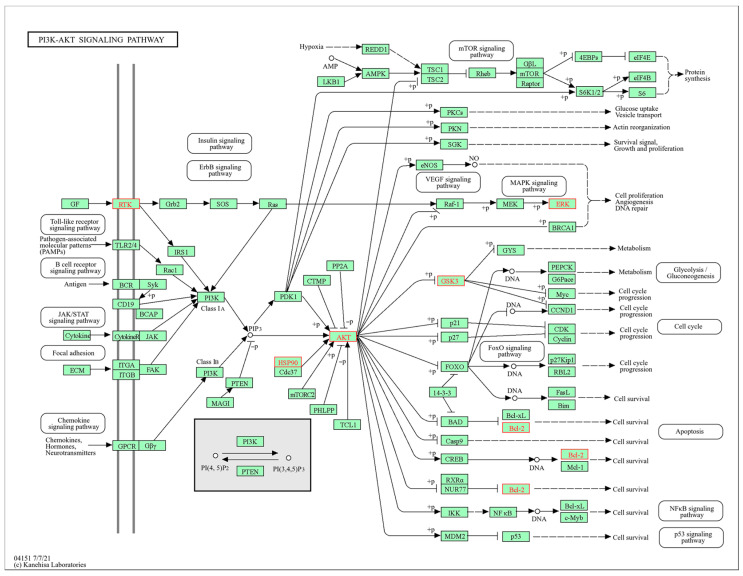
PI3K/AKT signaling pathway.

A total of 144 potential target genes were uploaded to the DAVID database for GO and KEGG pathway analysis. The GO enrichment analysis included biological processes (BP), cellular components (CC), and molecular functions (MF). The results indicated the presence of 496 BPs, 73 CCs, and 138 MFs. The top 10 degrees for each category were selected to create histograms (Figure 10A). BP enrichment primarily involved responses to external stimuli, negative regulation of apoptosis processes, and glucose homeostasis. CC enrichment was mainly observed in the plasma membrane, receptor complexes, and cytoplasm. MF enrichment primarily related to RNA polymerase II transcription factor activity, steroid binding, and enzyme binding, among others. In addition, KEGG enrichment analysis identified 153 signaling pathways, and the top 20 pathways with the highest degree values were selected for analysis, represented using a bubble chart (Figure 10B). These pathways include the EGFR tyrosine kinase inhibitor resistance signaling pathway, signaling pathways in cancer, insulin resistance signaling pathway, and chemical carcinogen–receptor activation signaling pathway, among others. The primary signaling pathway involved in glucose metabolism and the induction of diabetes is the PI3K/AKT signaling pathway (Figure 11). The improving effect of SD on diabetes may be mediated through this signaling pathway.

#### 2.6.4. Construction of the “C-T-P” Network Diagram

**Figure 12:**
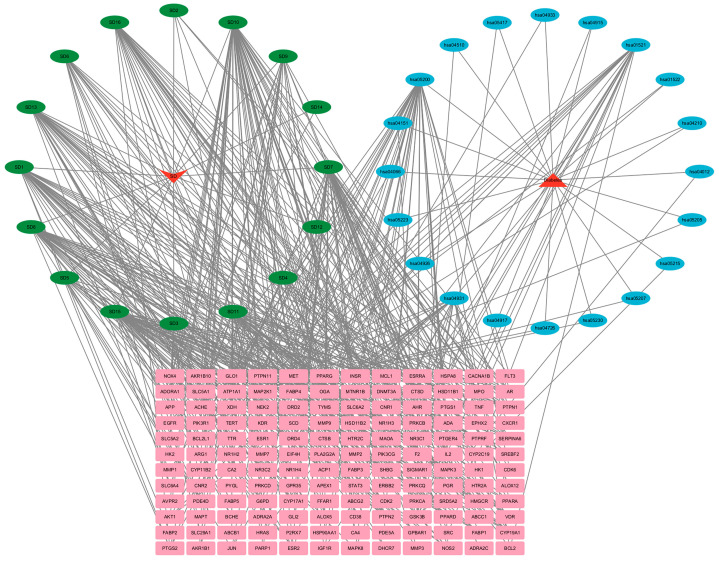
The “Compound-Target-Pathway” network. Green ovals represent compounds, blue ovals represent signaling pathways, and pink rectangles represent signaling pathways.

To clarify the connections between compounds, potential therapeutic targets, and the top 20 signaling pathways, a “C-T-P” network was constructed. The three compounds most closely associated with potential therapeutic targets in SD are quercetin (target number = 52), taraxerol (target number = 38), and spinasterol (target number = 37). Figure 12 reveals the interactions among 16 compounds, 144 targets, and 20 signaling pathways, with denser lines indicating more pronounced interactions.

### 2.7. Molecular Docking Analysis

Among the top 10 core targets for SD treatment of DM, AKT1 (PDB ID: 7NH5), BCL2 (PDB ID: 5JSN), and STAT3 (PDB ID: 6TLC) with the highest degree values were selected for molecular docking with five potential active components: quercetin, taraxerol, spinasterol, isoquercitrin, and syringin (Table S6). To explore the binding modes of the potential active components with these three targets, we utilized MOE to predict their interactions. The binding energy was assessed to evaluate the interaction strength between the small molecules and the protein targets. Generally, a binding energy less than 0 kcal mol^−1^ indicates that there is binding activity between the molecules, while a binding energy less than −5 kcal mol^−1^ indicates a strong binding activity. The smaller the binding energy, the stronger the binding affinity.

**Figure 13:**
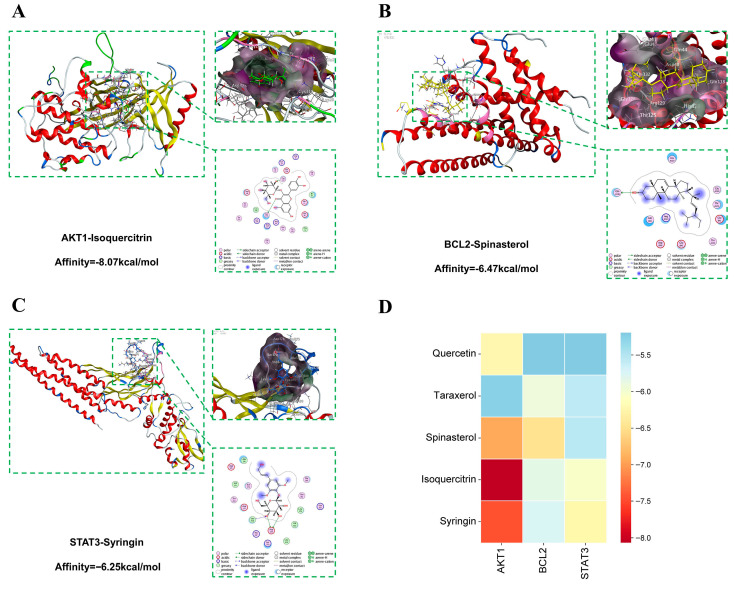
The 2D and 3D schematic diagrams of molecular docking sites and heat maps of molecular docking results. (**A**) AKT1-Isoquercitrin; (**B**) BCL2-Spinasterol; (**C**) STAT3-Syringin; (**D**) heatmap of docking results.

Quercetin, taraxerol, spinasterol, isoquercitrin, and syringin all exhibit binding energies less than −5 kcal mol^−1^ with key targets, indicating favorable docking potential (Figure 13D). Among these, hydrogen bonds and hydrophobic interactions play a crucial role in the binding energy. As shown in Figure 13A–C, the binding energy between isoquercitrin and AKT1 is −8.07 kcal mol^−1^, where it forms hydrogen bonds with the CYS296 and CYS310 residues of AKT1 and engages in hydrophobic interactions with several amino acid residues. The binding energy of spinasterol with BCL2 is −6.47 kcal mol^−1^, forming hydrogen bonds with the GLN118 residue of BCL2 and hydrophobic interactions with several amino acid residues. Syringin demonstrates a binding energy of −6.25 kcal mol^−1^ with STAT3, forming hydrogen bonds with the ASP369 and LEU438 residues of STAT3 and engaging in hydrophobic interactions with several amino acid residues.

### 2.8. The Inhibitory Effect of Potential Bioactive Components from SD on α-Glucosidase

**Figure 14:**
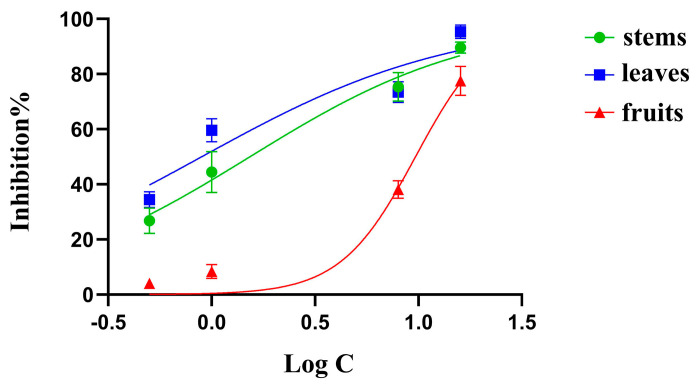
Dose-response curves for different regions of SD.

The α-glucosidase inhibitory activity of the crude extracts from different parts of SD were tested. The results indicated that the leaf extract of SD had the strongest α-glucosidase inhibitory capability, with an IC_50_ value of 0.90 ± 0.16 mg/mL. This was followed by the stem extract with an IC_50_ value of 1.52 ± 0.34 mg/mL. In contrast, the fruit extract exhibited the weakest inhibitory activity, with an IC_50_ value of 9.73 ± 0.70 mg/mL (Table 1, Figure 14).

**Figure 15:**
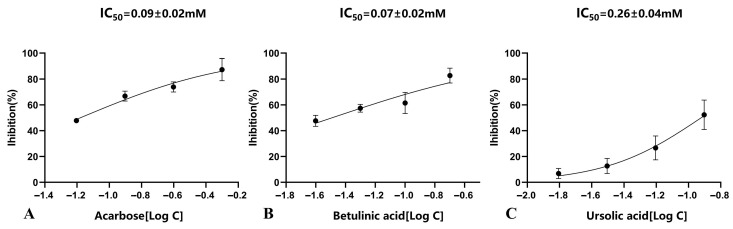
IC_50_ regression fitting curve analysis. (**A**) Positive control Acarbose. (**B**) Betulinic acid. (**C**) Ursolic acid.

The potential hypoglycemic core compounds identified through network pharmacology and compounds previously isolated from SD in our laboratory that may exhibit hypoglycemic activity were tested for their inhibitory activity against α-glucosidase using the PNPG method [31]. The results showed that betulinic acid exhibited the strongest inhibitory ability against α-glucosidase, with an IC_50_ value of 0.07 ± 0.02 mM, which is half that of the positive drug acarbose; followed by ursolic acid, with an IC_50_ value of 0.26 ± 0.04 mM. Squalene demonstrated the weakest inhibitory activity, with an IC_50_ value of 55.08 ± 1.80 mM (Table 2). The fitting curves for betulinic acid, ursolic acid, and the positive drug acarbose are shown in Figure 15.

## 3. Discussion

In this study, we conducted a comparative analysis of the secondary metabolites in the stems, leaves, and fruits (S, L, F) of *S. dulcificum* using widely targeted metabolomics based on UPLC-MS/MS. A total of 2544 secondary metabolites were identified, primarily including terpenes, phenolic acids, flavonoids, alkaloids, lignans, amino acids and derivatives, lipids, organic acids, coumarins, nucleotides and derivatives, tannins, quinones, and steroids. Among them, phenolic acids and flavonoids were major secondary metabolites. For flavonoids, 45 flavonoid, 26 flavonols, 17 flavonoid carbonoside, 12 anthocyanins, 9 dihydroflavone, 6 dihydroflavonol, and 3 flavanols were present in *S. dulcificum*. Some of the highly accumulated flavonoids were shown to possess anti-diabetic bioactivities and other health benefits, including quercetin, isoquercitrin, and rutin [32]. In addition, quercetin also has a strong effect in terms of antioxidant activity [33]. For example, quercetin affects many factors and signaling pathways related to insulin resistance and the pathogenesis of type 2 diabetes, demonstrating significant effects in the prevention and treatment of diabetic complications [34]. Isoquercitrin plays an important role in regulating STAT3 to alleviate diabetic nephropathy [35].

Among these, the content of lignans and coumarins was higher in S; L displayed a richer variety of flavonoids. The difference in the metabolites may be the reason why the leaves of *S. dulcificum* exhibit strong antibacterial and antioxidant activities [36,37]. In addition, F contained more alkaloids and lipids. This study analyzed and identified various categories of secondary metabolites in the stems, leaves, and fruits of *S. dulcificum*, providing a valuable reference for the isolation and identification of functional compounds present in the plant.

In the analysis of network pharmacology, 144 disease targets related to diabetes were identified, and 16 secondary metabolites with favorable pharmacokinetic properties were selected, leading to the construction of a PPI network and a “C-T-P” network. From this, 10 key targets with the highest network connectivity (STAT3, BCL2, AKT1, ESR1, EGFR, HSP90AA1, MMP9, SRC, TNF, JUN) were chosen to create a core network diagram, indicating that they may play a central role in the treatment of diabetes.

KEGG pathway enrichment analysis indicated that the anti-diabetic effects of *S. dulcificum* may be mediated through various signaling pathways, including the P13K/AKT, EFGR, mTOR, and insulin resistance pathways. Among these, the P13K/AKT signaling pathway plays a critical role in regulating glucose metabolism and is a key pathway in diabetes treatment. This pathway is essential for insulin signal transduction, promoting glucose uptake, and maintaining glucose homeostasis. The P13K/AKT signaling pathway can positively regulate insulin expression and inhibit β-cell apoptosis, thereby improving insulin quality and function in diabetic rats [38]. Furthermore, the activation of the P13K/AKT signaling pathway helps to improve the disruption of glucose and lipid metabolism in the body and contributes to the maintenance of blood glucose balance [39].

Additionally, the three disease targets with the highest connectivity values in the string network, STAT3, BCL2, and AKT1, also mediate multiple diabetes-related signaling pathways and are key targets in the treatment of diabetes. Signal transducer and activator of transcription 3 (STAT3) is a key transcription factor closely associated with chronic inflammation, autoimmune diseases, and tumor development [40]. Research has shown that STAT3 can regulate diabetes by controlling hepatic glucose metabolism, including gluconeogenesis and glycolysis, as well as insulin sensitivity [41]. The liver-specific knockout of STAT3 negatively regulates gluconeogenesis, increasing the expression of hepatic gluconeogenesis genes. Conversely, liver-specific activation of STAT3 lowers blood glucose levels, plasma insulin levels, and the expression levels of gluconeogenesis genes Pck1 and G6pc in diabetic mice [42].

The B cell lymphoma 2 (BCL2) protein family plays a crucial role in regulating apoptosis by controlling the release of cytochrome c from mitochondria, thereby modulating cell death. Dysregulation of the BCL2 protein family is associated with various diseases, including cancer, neurodegenerative diseases, and autoimmune disorders [43]. It has been reported that MSCs exhibit their anti-apoptotic effects by downregulating ROS, caspase 3, caspase 8, and p53, while upregulating Bcl2, thereby enhancing insulin secretion rates and promoting insulin regeneration [44].

Protein kinase B (AKT) regulates various cellular physiological functions upon activation, affecting multiple processes such as cell proliferation, survival, metabolism, and angiogenesis. Its regulatory mechanisms involve the interaction of multiple signaling axes, including the PI3K/AKT/mTOR and RAS/ERK pathways [45]. Research has found that gastrin-SiO_2_ microspheres reduce intestinal glucose absorption by downregulating the expression of SGLT1 and GLUT2 and stimulating the secretion of incretins. The gastrin/CCKBR axis inhibits the expression of SGLT1 and GLUT2 through the PI3K/AKT/eIF4B signaling pathway, thereby preventing type 2 diabetes [46]. Overall, the signal pathway enrichment analysis provides strong evidence that the bioactive components of *S. dulcificum* target various pathways and molecular mechanisms related to diabetes, enhancing its potential as a natural therapeutic agent. Future experimental validation through in vitro and in vivo studies is crucial to further confirm these computational results and establish specific molecular mechanisms.

In molecular docking simulations, *S. dulcificum* bioactive compounds isoquercitrin, spinasterol, and syringin, which exhibit favorable pharmacokinetic properties, showed strong interactions with three key targets: AKT1, BCL2, and STAT3, respectively. The binding energies were all below −4.5 kcal/mol, indicating the potential to form stable complexes. The AKT1–isoquercitrin complex exhibited strong hydrogen bonds and van der Waals interactions, suggesting its potential role in regulating the PI3K/AKT pathway. The BCL2–spinasterol complex demonstrated significant non-covalent interactions, which may influence BCL2′s control over the apoptosis process. Similarly, the STAT3–syringin complex exhibited strong hydrogen bonds and van der Waals interactions, indicating its potential impact on STAT3-mediated glucose and lipid metabolism processes.

In the in vitro α-glucosidase inhibition experiment, betulinic acid and ursolic acid exhibited strong inhibitory activity, particularly ursolic acid, which showed an IC_50_ value against α-glucosidase that was only half that of the positive control acarbose, demonstrating significant potential for diabetes treatment. Betulinic acid is a penta-cyclic triterpenoid compound widely present in the plant kingdom, and it has garnered attention due to its activities such as anti-tumor, anti-parasitic, anti-HIV, anti-inflammatory, anti-diabetic, and pro-apoptotic effects [47]. According to reports, betulinic acid, as a PPARγ antagonist, can inhibit diabetes by promoting osteogenic differentiation and suppressing adipogenesis [48,49]. At the same time, betulinic acid activates AMPK, thereby reducing the expression of phosphoenolpyruvate carboxy kinase and glucose-6-phosphatase. It also increases transmembrane glucose transport and promotes the expression of glucose transporters GLUT-1 and GLUT-2, facilitating glucose uptake and glycogen synthesis [50,51]. Ursolic acid is a triterpenoid compound with a pentacyclic structure, exhibiting various biological activities such as sedative, anti-inflammatory, antibacterial, and anticancer effects, among which its cardiovascular-protective and anti-diabetic properties have attracted particular attention [52]. Ursolic acid enhances insulin signaling and achieves hypoglycemic effects by increasing the phosphorylation of the insulin receptor beta (IRβ) sub-unit and activating the PI3K/AKT signaling pathway. The activated PI3K and AKT promote the fusion of glucose transporter 4 (GLUT4) with the cell membrane, thereby increasing the uptake of glucose at the membrane surface [53]. In enzyme inhibition experiments, compounds with potent inhibitory effects are mainly flavonoids and pentacyclic triterpenes. A representative compound, ursolic acid, directly participates in insulin signaling pathways to lower blood sugar and exerts anti-diabetic effects. On the other hand, betulinic acid indirectly influences the expression of diabetes-related signaling pathways, promoting glucose uptake and glycogen synthesis, thereby displaying anti-diabetic effects. Notably, compared to stems and fruits, *S. dulcificum* leaves contain a comparatively higher content of flavonoids and pentacyclic triterpenes, suggesting the leaves may possess greater anti-diabetic potential. The above evidence indicates that certain secondary metabolites in *S. dulcificum* have significant anti-diabetic potential, providing an important theoretical basis for the future development of diabetes treatments.

## 4. Conclusions

This study is the first to use broad-target metabolomics technology based on UPLC-MS/MS to identify metabolites in the stems, leaves, and fruits of *S. dulcificum*, investigating the metabolic differences and the composition of functional compounds among these tissues. Additionally, through the integration of network pharmacology, molecular docking, and in vitro bioactivity experiments, a theoretical foundation for the anti-diabetic mechanisms of *S. dulcificum* was established. However, there is a lack of in vivo and in vitro studies to validate these findings, which are critical for assessing their true physiological effects. Overall, this study enhances our understanding of the exploration of *S. dulcificum* secondary metabolites and their anti-diabetic mechanisms, providing valuable insights for future drug development.

## 5. Materials and Methods

### 5.1. Materials

The healthy plant stems, leaves, and fruits of SD were collected from the same plantation in Xishuangbanna Prefecture (21°8′–22°36′ N, 99°56′–101°51′ E), Yunnan, China (Figure S1). All SD samples were certified by researcher Shaohua Wu from Yunnan University.

Ultrapure water was obtained using the UP series ultrapure water system (Chengdu, China). The reference standards, including betulinic acid (98%), syringin (98%), stigmasterol (98%), squalene (98%), quercetin (98%), isoquercitrin (98%), and α-glucosidase (yeast sources), were obtained from Nanjing Yuanzhi Biotechnology Co., Ltd. (Nanjing, China). Para-nitrophenyl-α-D-galactopyranoside (PNPG) and acarbose were purchased from Shanghai Yuanye Biotechnology Co., Ltd. (Shanghai, China). Ursolic acid, corosolic acid, and methyl gallate were isolated and obtained from the leaves of the SD in our previous experiments, and preserved at the Yunnan Institute of Microbiology [31]. HPLC-grade formic acid was purchased from Shanghai Aladdin Biochemical Technology Co., Ltd. (Shanghai, China). HPLC-grade methanol and acetonitrile were purchased from Merck (Darmstadt, Germany). Di-methyl sulfoxide (DMSO) was purchased from Tianjin Dama Chemical Reagent Factory (Tianjin, China). Disodium hydrogen phosphate was purchased from Guangdong Guanghua Technology Co., Ltd. (Shantou, China). Other analytical-grade chemicals and reagents, such as sodium chloride and anhydrous ethanol, were obtained from Sichuan Xilong Scientific Co., Ltd. (Chengdu, China).

### 5.2. Widely-Targeted Metabolomic Analysis of Secondary Metabolites

#### 5.2.1. Sample Preparation and Extraction

Using vacuum freeze-drying technology, place the biological samples in a lyophilizer (Scientz-100F), then grind (30 Hz, 1.5 min) the samples to powder form by using a grinder (MM 400, Retsch, Haan, Germany) Next, weigh 50 mg of sample powder using an electronic balance (MS105DΜ) and add 1200 μL of −20 °C pre-cooled 70% methanolic aqueous internal standard extract (less than 50 mg added at the rate of 1200 μL extractant per 50 mg sample). Vortex once every 30 min for 30 sec, for a total of 6 times After centrifugation (rotation speed 12,000 rpm, 3 min). The supernatant was aspirated, and the sample was filtered through a microporous membrane (0.22 μm pore size) and stored in the injection vial for UPLC-MS/MS analysis.

#### 5.2.2. LC-MS/MS Conditions

The sample extracts were analyzed using an UPLC-ESI-MS/MS system (UPLC, ExionLC™ AD https://sciex.com.cn/, accessed on 2 November 2024) and Tandem mass spectrometry system (https://sciex.com.cn/, accessed on 2 November 2024). The analytical conditions were as follows, UPLC: column, Agilent, Santa Clara, CA, USA. SB-C18 (1.8 µm, 2.1 × 100 mm); the mobile phase consisted of solvent A, pure water with 0.1% formic acid, and solvent B, acetonitrile with 0.1% formic acid. Sample measurements were performed with a gradient program that employed the starting conditions of 95% A, 5% B. Within 9 min, a linear gradient to 5% A, 95% B was programmed, and a composition of 5% A, 95% B was kept for 1 min. Subsequently, a composition of 95% A, 5.0% B was adjusted within 1.1 min and kept for 2.9 min. The flow velocity was set as 0.35 mL per minute; the column oven was set to 40 °C; the injection volume was 2 μL. The effluent was alternatively connected to an ESI-triple quadrupole-linear ion trap (QTRAP)-MS (Agilent, Santa Clara, CA, USA).

The ESI source operation parameters were as follows: source temperature 500 °C; ion spray voltage (IS) 5500 V (positive ion mode)/−4500 V (negative ion mode); ion source gas I (GSI), gas II(GSII), curtain gas (CUR) were set at 50, 60, and 25 psi, respectively; the collision-activated dissociation (CAD) was high. QQQ scans were acquired as MRM experiments with collision gas (nitrogen) set to medium. DP (declustering potential) and CE (collision energy) for individual MRM transitions was carried out with further DP and CE optimization. A specific set of MRM transitions was monitored for each period according to the metabolites eluted within this period.

#### 5.2.3. Qualitative and Quantitative Analysis of Secondary Metabolites

Based on the self-built database MWDB (Metware Biotechnology Co., Ltd. Wuhan, China), qualitative analysis of substances was conducted according to secondary spectral information. Isotope signals, as well as overlapping signals from ions such as K+, Na+, and NH4+, were removed during analysis, along with repetitive signal fragments from larger molecular weight substances. Quantification of metabolites was achieved using multiple reaction monitoring (MRM) mode of triple quadrupole mass spectrometry. In the MRM mode, the first quadrupole filters the precursor ions (parent ions) of the target substances, excluding ions corresponding to other molecular weight substances to minimize interference. The precursor ions are then induced to fragment in the collision chamber, generating multiple fragment ions. These fragment ions are subsequently filtered in the triple quadrupole to select a specific characteristic fragment ion, thereby eliminating non-target ion interference, which enhances the precision and reproducibility of quantification. After obtaining mass spectrometric analysis data of metabolites from different samples, peak area integration of all chromatographic peaks was performed, and integration correction of the mass spectrometric peaks for the same metabolite across different samples was carried out [54].

### 5.3. Network Pharmacology

#### 5.3.1. Screening for Potential Active Ingredients

The Traditional Chinese Medicine System Pharmacology Database and Analysis Platform (TCMSP, https://www.tcmsp-e.com/#/database, accessed on 11 July 2024) is a recognized pharmacological platform for herbal medicine, used to retrieve the relationships between drug components, targets, and diseases [55]. Based on the relevant drug screening criteria (OB ≥ 30%, DL ≥ 0.18) suggested by the TCMSP database [56], all secondary metabolites identified in broad-targeted metabolomics were screened to find potential active ingredients. At the same time, pharmacokinetic parameters of all secondary metabolites were evaluated using the online servers pkCSM (https://biosig.lab.uq.edu.au/pkcsm/prediction, accessed on 11 July 2024) and Swiss ADME (http://www.swissadme.ch/, accessed on 11 July 2024) to ensure they meet ADMET standards and the five Lipinski rules. Although some components, such as syringin and isoquercitrin, do not meet the screening criteria, related studies have reported that they exhibit certain hypoglycemic activities [57,58,59]; therefore, they are also classified as potential effective ingredients.

#### 5.3.2. Prediction of Potential Active Ingredients and Diabetes-Related Targets

The target genes corresponding to the potential active components were obtained from the online platforms Swiss Target Prediction (http://www.swisstargetprediction.ch/, accessed on 14 July 2024, Probability > 0.1,) and PubChem (https://pubchem.ncbi.nlm.nih.gov/, accessed on 14 July 2024). The diabetes-related target genes were identified by searching the keyword “diabetes mellitus” in the GeneCard (https://www.genecards.org/, accessed on 16 July 2024, Score > 0.1) and DisGeNET (https://www.disgenet.org/search, accessed on 16 July 2024, Score > 0) online databases. Additionally, we downloaded three diabetes-related microarray datasets, GSE21340, GSE29221, and GSE55650, from the GEO database. Detailed information about all GSE microarray datasets is provided in Table 3. Using the R programming language version 4.2.2 and the R 4.2.2 package limma, we identified differentially expressed genes (DEGs) in each GSE dataset, obtaining both upregulated and downregulated genes associated with diabetes. Finally, we integrated the disease genes identified through database searches with the upregulated genes from the GSE datasets, removing duplicates.

#### 5.3.3. Construction of Drug–Compound–Target Networks and Pathway Analysis

A drug–compound–target interaction network was constructed using Cytoscape software (version 3.10.0). Gene Ontology (GO) functional annotation and Kyoto Encyclopedia of Genes and Genomes (KEGG) pathway enrichment analysis were performed using the DAVID database platform (https://david.ncifcrf.gov/), selecting “homo sapiens” for species. These pathways were visualized using bubble plots and histograms through an online bioinformatics platform [60].

#### 5.3.4. The Construction of Protein–Protein Interaction (PPI) Network and Component–Target–Disease (C–T–P) Network

Using the STRING database (https://cn.string-db.org/, accessed on 17 July 2024) to explore potential interactions between candidate therapeutic targets, the parameter settings are as follows: the species is set to “Homo sapiens,” with a minimum interaction threshold greater than 0.4, and nodes that are not connected to the network are removed. The resulting PPI network is analyzed and visualized using Cytoscape 3.10.0. The CytoNCA plugin is utilized to calculate node topology parameters, identifying core targets with high Degree values. The C–T–P network is constructed by importing data on active compounds, potential therapeutic targets, and the top 20 signaling pathways into Cytoscape 3.10.0, with core compounds screened based on degree values in the network [61].

#### 5.3.5. Molecular Docking Verification

Molecular docking, a crucial technique in network pharmacology analysis, involves combining known protein targets with small compounds to validate compound–target interactions [62]. The top three target proteins with the highest CytoNCA scores from the core PPI network were selected as receptors, and the top five potential active compounds with the highest degree values from the C–T–P network were chosen as ligands. This study utilized the 2019 version of the Molecular Operating Environment (MOE) software for molecular docking analysis. The 3D format of the core targets was obtained from the Protein Data Bank (PDB) (https://www.rcsb.org/, accessed on 16 September 2024), AKT1 (PDB ID: 7NH5), BCL2 (PDB ID: 5JSN), and STAT3 (PDB ID: 6TLC), followed by preprocessing steps including removal of water molecules, preparation of the protein structure, and energy minimization. The structures of active compounds were sourced from Pub-Chem (https://pubchem.ncbi.nlm.nih.gov/, accessed on 16 September 2024), and then both the protein structure and the compound structures were imported into MOE to construct the docking interface, enabling molecular docking analysis.

### 5.4. In Vitro Inhibitory Activity Against α-Glucosidase

Soak the crushed stems, leaves, and fruits of *S. dulcificum* in methanol for 24 h, then extract the chemical components using ethyl acetate. After that, concentrate the extract using a rotary vacuum evaporator, repeating the extraction three times to obtain the crude extracts, which are then prepared at concentrations of 0.5, 1, 8, and 16 mg/mL, respectively, for subsequent inhibition experiments.

The assay was conducted following a previously described method [63], and made some minor adjustments. Briefly, 0.1 M phosphate-buffered saline (PBS, pH 6.9) was used to prepare standard sample solutions of different concentrations, α-glucosidase (1 U/mL), and p-nitrophenyl-α-D-glucopyranoside (α-PNPG, 5 mM). A mixture of the sample (50 μL) and α-glucosidase (50 μL) was incubated at 37 °C for 10 min, followed by the addition of 50 μL of PNPG and further incubation at 37 °C for 20 min. Finally, 100 μL of Na_2_CO_3_ (0.1 M) was added to terminate the reaction after incubating at 37 °C for 10 min. The mixture without the sample served as a negative control, while acarbose served as a positive control. The absorbance was measured using a microplate reader at 405 nm. The experiment was set up with three repetitions, and the IC50 values were determined. The method for determining α-glucosidase inhibitory activity is as follows:

(1) $$\mathrm{Inhibition}\%=\left(\frac{Absorbance_{Negative\;control}-Absorbance_{sample}}{Absorbance_{Negative\;control}}\right)\times 100\%$$

## Figures and Tables

**Table 1:** IC_50_ of α-glucosidase inhibition assay of *S. dulcificum* crude extract.

Different Parts of *S. dulcificum*	Stems	Leaves	Fruits
IC_50_ (mg/mL)	1.52 ± 0.34	0.90 ± 0.16	9.73 ± 0.70

**Table 2:** IC_50_ values of α-glucosidase inhibition assay of *S. dulcificum* components.

Component	IC_50_ ± SD (mM)
Quercetin	1.26 ± 0.13
Betulinic acid	0.07 ± 0.02
Squalene	55.08 ± 1.80
Syringin	5.40 ± 1.20
Stigmasterol	2.11 ± 0.24
Ursolic acid	0.26 ± 0.04
Corosolic acid	2.12 ± 0.44
Methyl gallate	4.18 ± 1.09
Acarbose *	0.09 ± 0.02

* Acarbose as a positive control.

**Table 3:** Information of the three GSE datasets.

Dataset ID	Platforms	Sample Number	Control Sample Number	Disease Sample Number	Organism	Experiment Type	Attribute
GSE29221	GPL6947	24	12	12	*Homo sapiens*	Array	Test
GSE21340	GPL80	20	10	10	*Homo sapiens*	Array	Test
GSE55650	GPL570	23	11	12	*Homo sapiens*	Array	Test

## Data Availability

Data are contained within the article and Supplementary Material.

## Supplementary Materials

The following supporting information can be downloaded at: https://www.mdpi.com/article/10.3390/plants14142132/s1.
