# Supplementary material for: Combined Metabolomics and Network Pharmacology to Reveal Anti-Diabetic Mechanisms and Potential Pharmacological Components of Synsepalum dulcificum
Source: Plants (Basel). 2025 Jul 10;14(14):2132. doi: 10.3390/plants14142132 (PMC12299813; doi:10.3390/plants14142132)
Supplement: Supplementary file 1 [file plants-14-02132-s001.zip › Supplement images.pdf]

Supplementary Materials

Figure S1 Samples of the stem, leaves, and fruit of the *Synsepalum dulcificum.*

Figure S2 (A-B, E-F, I-J, M-N) Total ion current (TIC) profile of QC, S, L, F samples mass spectrometry analysis; (C-D, G-H, K-L, O-P) QC, S, L, F samples MRM metabolite detection multi-peak chart. (A, C, E, G, I, K, M, O) represent the positive ion mode, (B, D, F, H, J, L, N, P) in negative ion mode.

Table S6 Ranking table of the association degree between *Synsepalum dulcificum* active compounds and targets.

Fig. S1

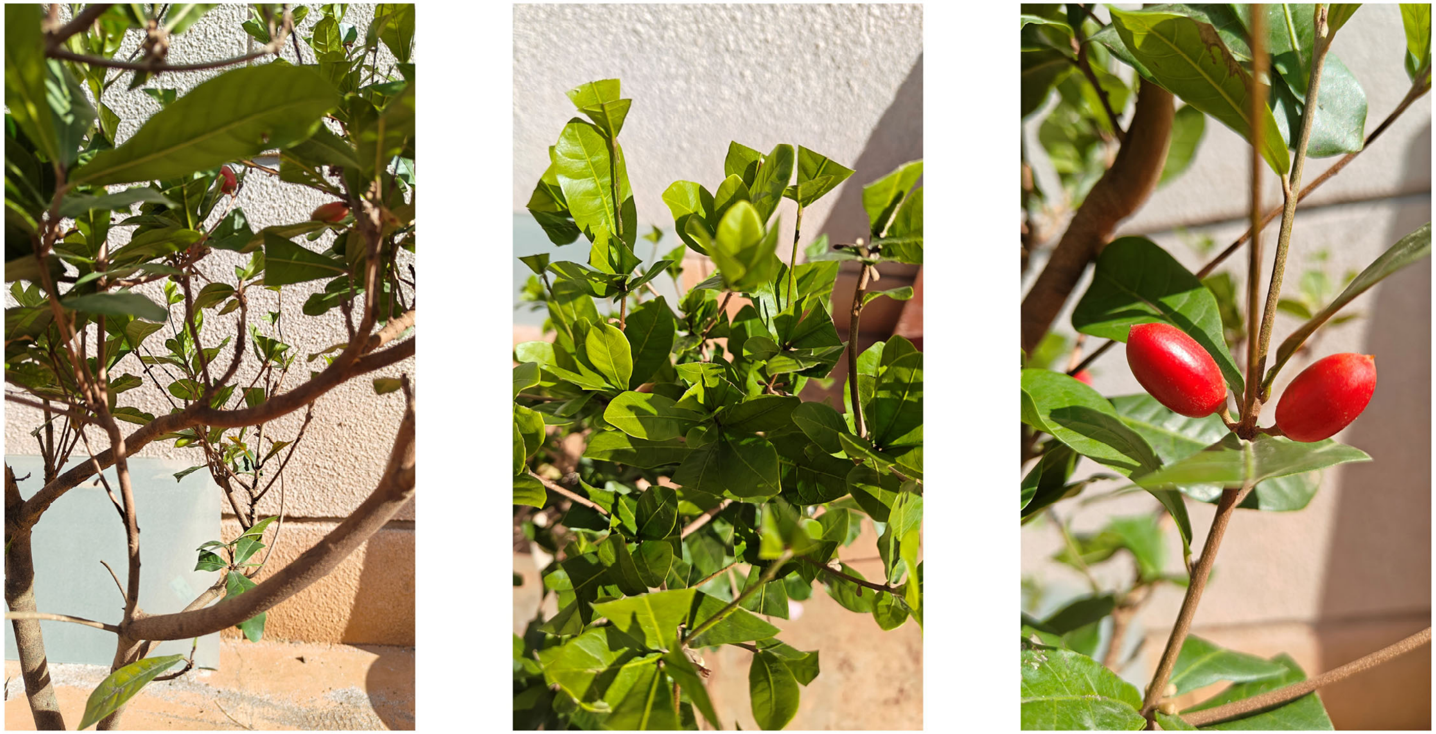

stem **leaves** **fruit**

Fig. S2 A-D

A

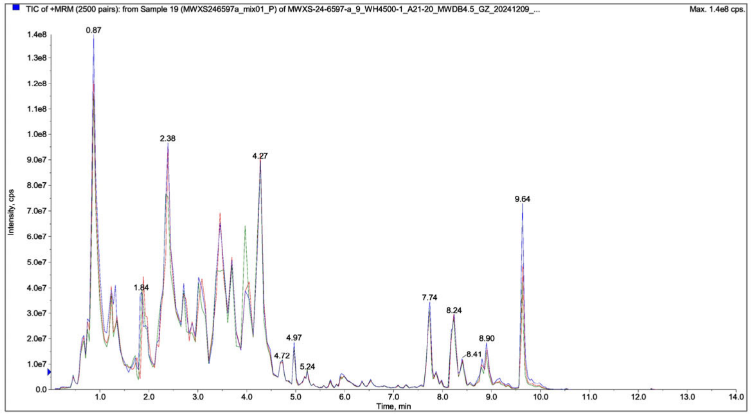

B

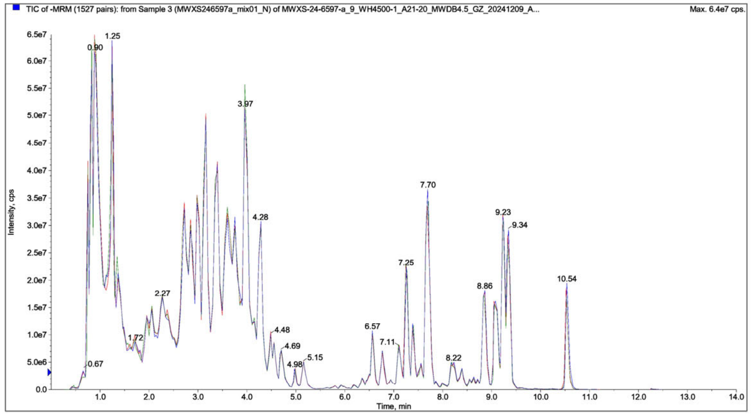

C

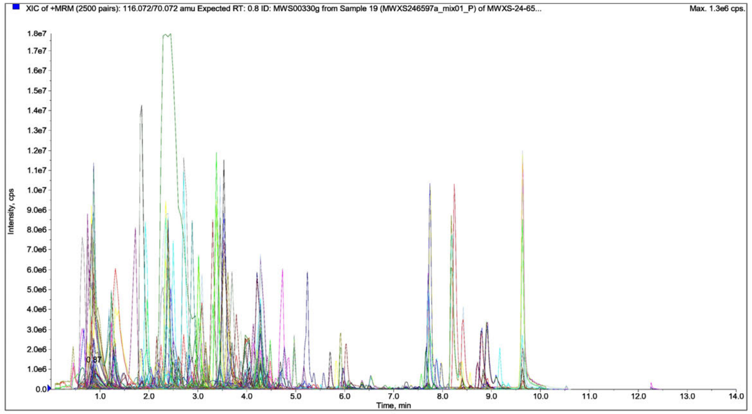

D

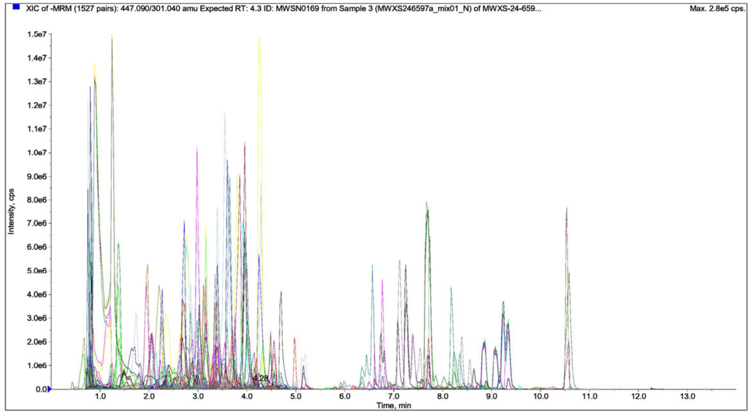

Fig. S2 E-H

E

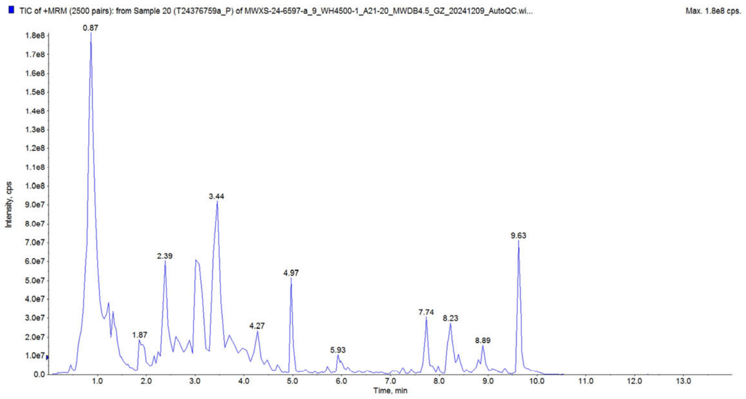

F

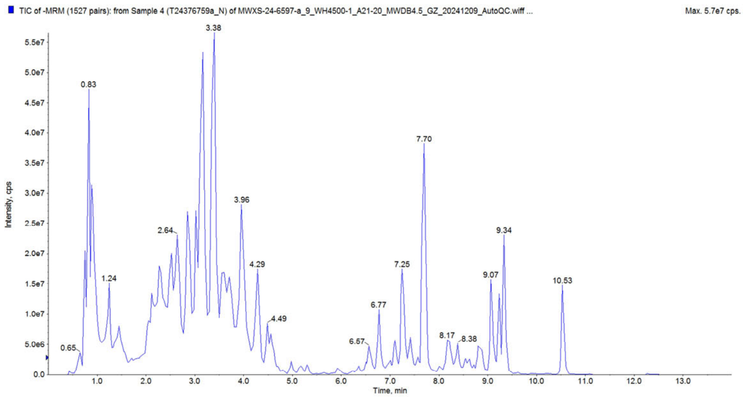

G

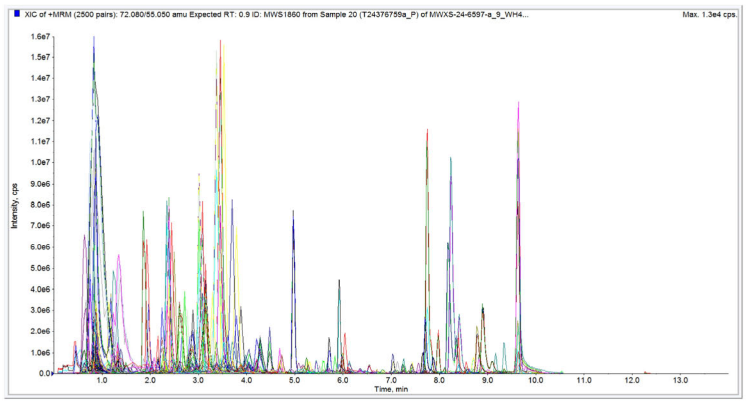

H

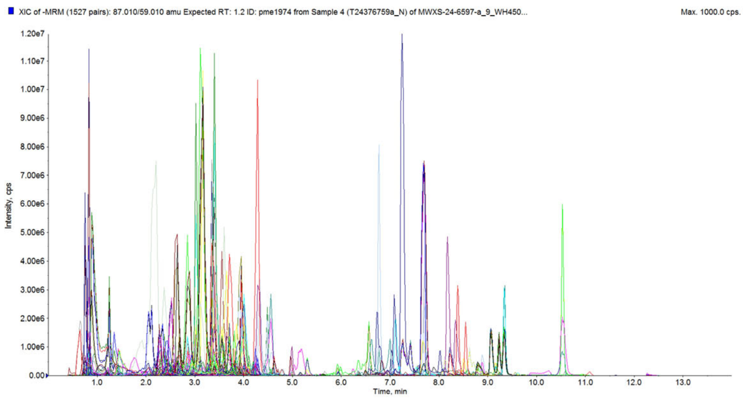

Fig. S2 I-L

I

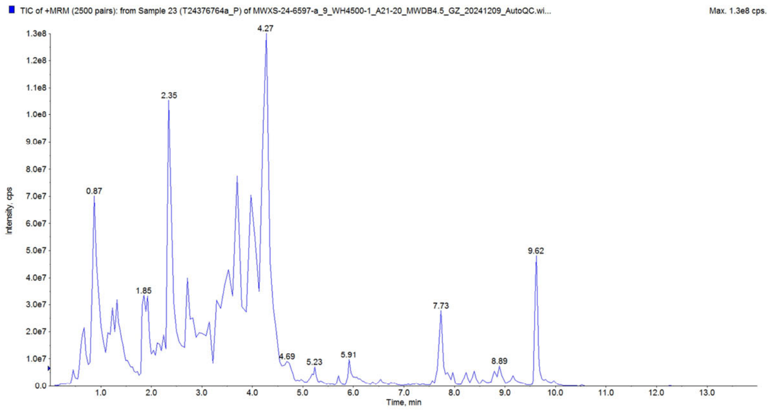

J

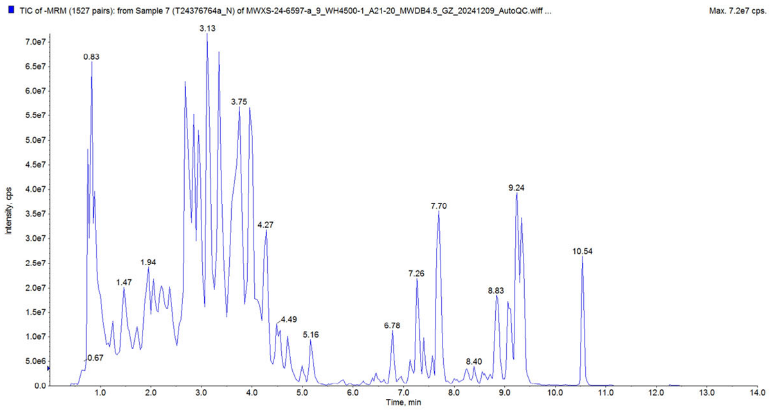

K

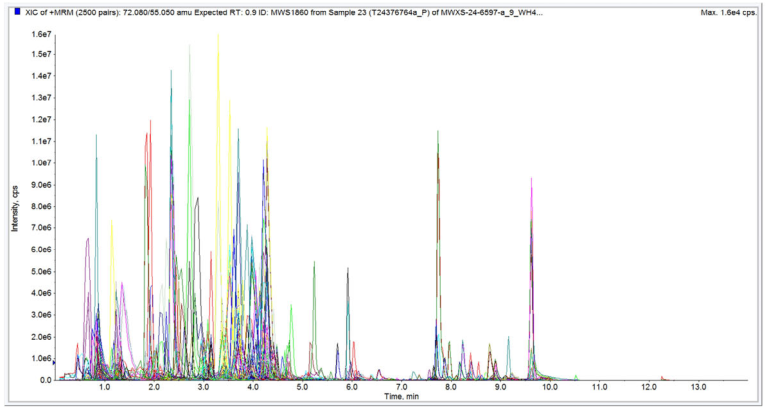

L

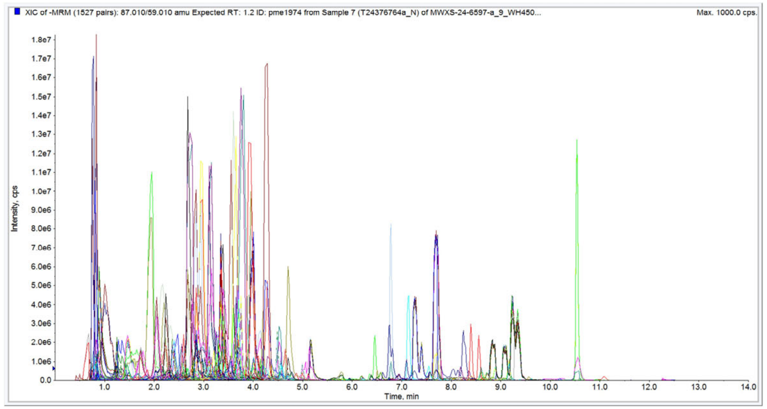

Fig. S2 M-P

M

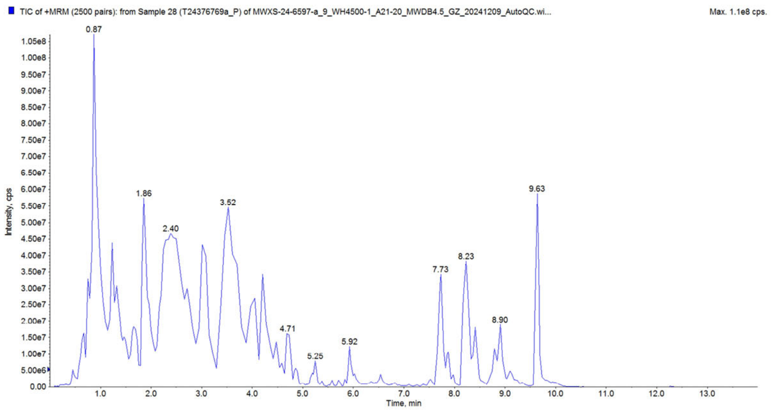

N

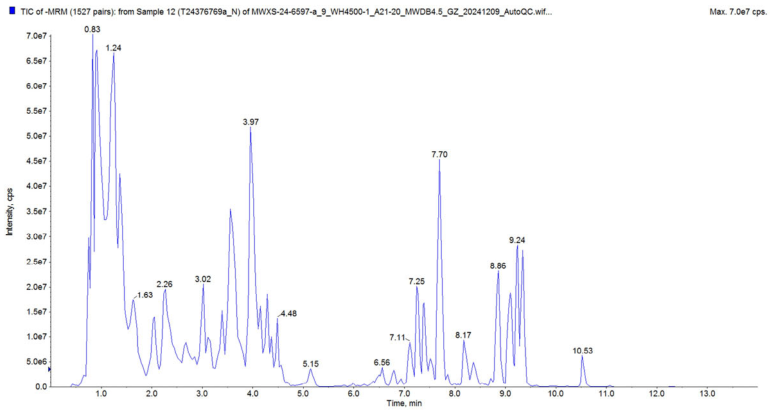

O

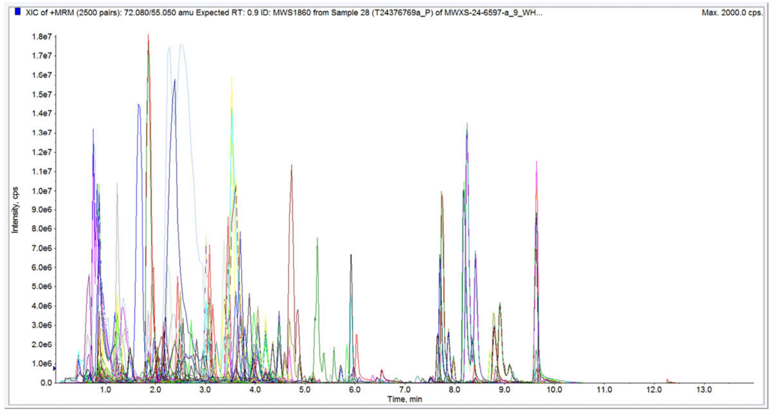

P

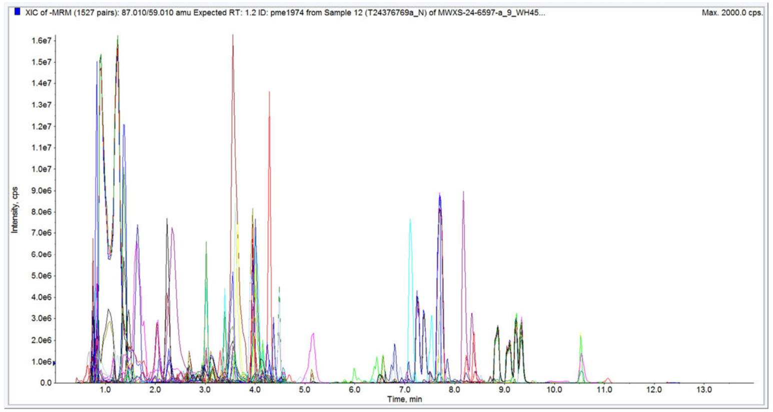

Table

CAS 号	化合物名称	Degree	结构式
117-39-5	Quercetin	52	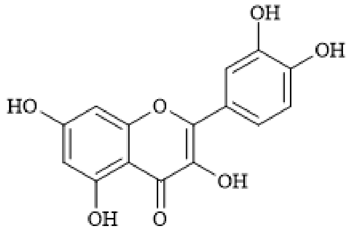
127-22-0	Taraxerol	38	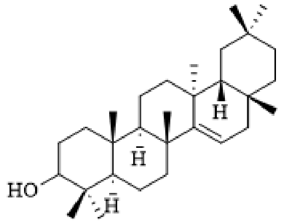
481-18-5	Spinasterol	37	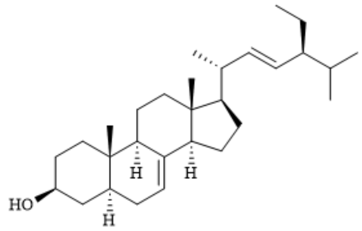
5896-2-6	Megastigmatrienone	36	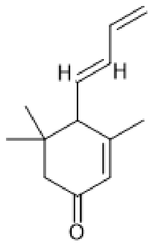
37905-13-8	Protobassic acid	34	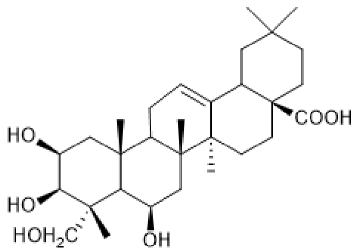
472-15-1	Betulinic acid	31	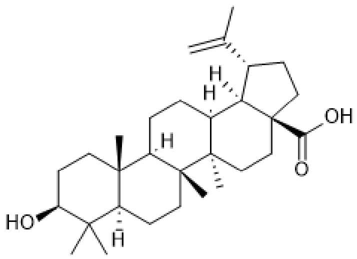

Continuation of the table.

CAS 号	化合物名称	Degree	结构式
83-48-7	Stigmasterol	27	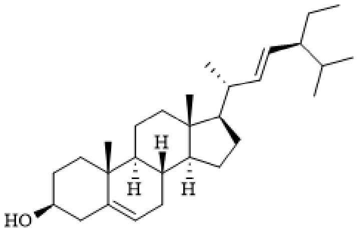
863-76-3	α-amyrin acetate	26	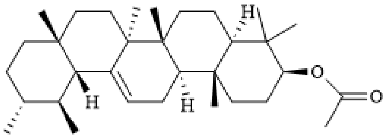
482-35-9	Isoquercetin	18	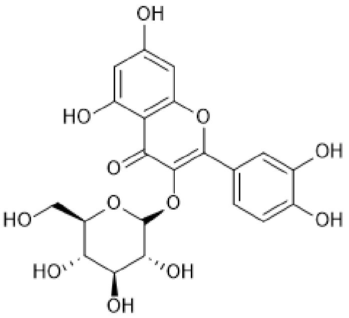
2400913-82-6	Maysedilactone C	15	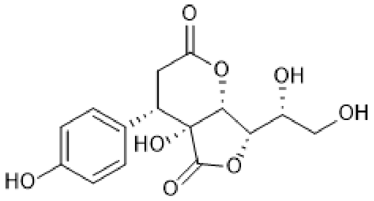
58019-21-9	Madlongiside D	13	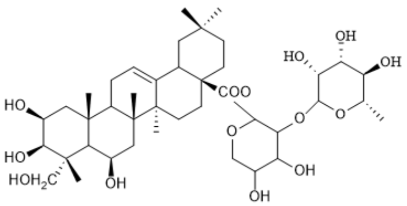
67258-70-2	Madlongiside C	10	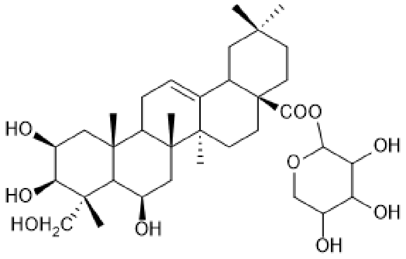

Continuation of the table.

CAS 号	化合物名称	Degree	结构式
195056-67-8	Junipetrioloside A	8	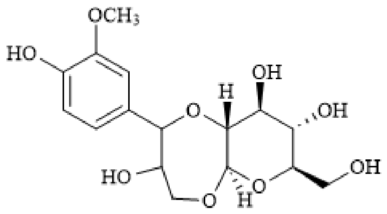
131-20-4	Diisooctyl phthalate	5	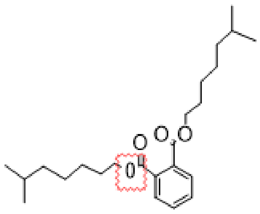
111-02-4	Squalene	3	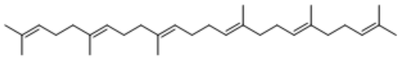
118-34-38	Syringin	2	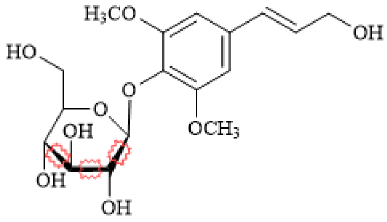
